# Recruitment of a Cytoplasmic Chaperone Relay by the A_2A_ Adenosine Receptor*

**DOI:** 10.1074/jbc.M113.464776

**Published:** 2013-08-21

**Authors:** Christian Bergmayr, Patrick Thurner, Simon Keuerleber, Oliver Kudlacek, Christian Nanoff, Michael Freissmuth, Christian W. Gruber

**Affiliations:** From the Institute for Pharmacology, Center for Physiology and Pharmacology, Medical University of Vienna, Waehringerstrasse 13a, A-1090 Vienna, Austria

**Keywords:** 7-Helix Receptor, Adenosine Receptor, Adenylate Cyclase (Adenylyl Cyclase), Endoplasmic Reticulum (ER), G Protein-coupled Receptors (GPCR), G Proteins, Heat-Shock Protein

## Abstract

The adenosine A_2A_ receptor is a prototypical rhodopsin-like G protein-coupled receptor but has several unique structural features, in particular a long C terminus (of >120 residues) devoid of a palmitoylation site. It is known to interact with several accessory proteins other than those canonically involved in signaling. However, it is evident that many more proteins must interact with the A_2A_ receptor, if the trafficking trajectory of the receptor is taken into account from its site of synthesis in the endoplasmic reticulum (ER) to its disposal by the lysosome. Affinity-tagged versions of the A_2A_ receptor were expressed in HEK293 cells to identify interacting partners residing in the ER by a proteomics approach based on tandem affinity purification. The receptor-protein complexes were purified in quantities sufficient for analysis by mass spectrometry. We identified molecular chaperones (heat-shock proteins HSP90α and HSP70-1A) that interact with and retain partially folded A_2A_ receptor prior to ER exit. Complex formation between the A_2A_ receptor and HSP90α (but not HSP90β) and HSP70-1A was confirmed by co-affinity precipitation. HSP90 inhibitors also enhanced surface expression of the receptor in PC12 cells, which endogenously express the A_2A_ receptor. Finally, proteins of the HSP relay machinery (*e.g.* HOP/HSC70-HSP90 organizing protein and P23/HSP90 co-chaperone) were recovered in complexes with the A_2A_ receptor. These observations are consistent with the proposed chaperone/coat protein complex II exchange model. This posits that cytosolic HSP proteins are sequentially recruited to folding intermediates of the A_2A_ receptor. Release of HSP90 is required prior to recruitment of coat protein complex II components. This prevents premature ER export of partially folded receptors.

## Teaser

**Background:** The A_2A_ receptor is known to accumulate in the endoplasmic reticulum.

**Results:** Mass spectrometry identified molecular chaperones (HSP90 and HSP70) bound to the A_2A_ receptor.

**Conclusion:** Sequential recruitment of chaperones to the cytosolic face of the A_2A_ receptor is consistent with a heat-shock protein relay assisting folding.

**Significance:** The observations are consistent with a chaperone/COPII exchange model, where heat-shock proteins bound to the receptor preclude its premature ER export.

## Introduction

In the past 15 years, it has been appreciated that G protein-coupled receptors (GPCRs)^3^ bind many additional proteins; these are being referred to as GPCR-interacting proteins or accessory proteins for lack of a better description. In contrast to many other GPCRs and, in particular, to the other members of the adenosine receptor family (1), the A_2A_ receptor has an unusually long intracellular C-terminal tail (122 amino acids of the human A_2A_ receptor compared with only 34 residues in the C terminus of the human A_1_ receptor). The juxtamembrane segment immediately adjacent to the seventh transmembrane helix is required for proper folding of the receptor. The rest of the C terminus (∼100 amino acids) is dispensable for ligand binding (2) and for G protein coupling (3). The A_2A_ receptor does not carry a cysteine residue at the end of the C-terminal helix 8, which, in the vast majority of rhodopsin-like GPCRs, is the site of palmitoylation (4). This suggests that the C terminus of the A_2A_ receptor is not constrained by a lipid anchor and is hence more flexible (5). These two features, relative length and flexibility, may combine to afford the interaction of the A_2A_ receptor with many additional accessory proteins other than G proteins, arrestins, and G protein receptor kinases. In recent years, several interaction partners were identified in yeast two-hybrid interaction screens, using the C terminus of the A_2A_ receptor as bait, and the list is rapidly growing (6). Examples are ARNO, the guanine nucleotide exchange factor for ARF6 (7, 8); the deubiquitinating enzyme USP4 (9); translin-X-associated protein (10); and α-actinin (11). In addition, the A_2A_ receptor can form a heteromeric complex with at least one other GPCR, namely the D_2_ dopamine receptor (7, 12), and it can transactivate the neurotrophin receptors TrkA and TrkB (13).

Although the list of these A_2A_ receptor-interacting partners appears to be long, it is far from complete; from its birth in the endoplasmic reticulum (ER) until it reaches its site of degradation in the lysosome, the A_2A_ receptor is expected to meet many companion proteins. Several observations suggest that the A_2A_ receptor incurs a folding problem. In PC12 cells, where the receptor is endogenously expressed, a large fraction of the receptor is retained within the cell under basal conditions (14); expression levels in PC12 cells can be increased by blocking the proteasomal degradation in the ER (9). Similarly, heterologously expressed receptors accumulate in a complex with calnexin in ER-derived multilamellar bodies (15). Finally, overexpression of USP4 results in deubiquitination of ER-resident A_2A_ receptors, relaxes ER quality control, and promotes trafficking of the receptor to the cell surface, whereas down-regulation of USP4 has the opposite effect (9). USP4 engages the C terminus of the A_2A_ receptor; ER-resident chaperones, however, only contact the lumenal portions of the receptor. Accordingly, there must be components that sense the folding state and possibly assist the folding of the receptor on the cytosolic side. Based on these considerations, we recently proposed a chaperone/coat protein complex II (COPII)-exchange model. This model posits that one or several chaperones engage the C terminus of the partially folded A_2A_ receptor; they are released if the receptor attains a stable fold, which renders the C terminus accessible to components of the COPII coat. If folding of the receptor stalls, it is relayed to the ER-associated degradation machinery. This model explains how premature recruitment of the COPII coat and, hence, export of partially folded receptors can be obviated by sequential binding of proteins to the C terminus (4).

Here, we searched for evidence in support of this model by employing a strategy relying on tandem affinity purification (TAP) of the receptor. We identified an abundant complex comprising heat-shock protein 90 (HSP90) and the A_2A_ receptor-chaperone protein complex, confirmed its identity by co-affinity and co-immunoprecipitation, and verified its relevance by the use of inhibitors. We also provide evidence for the existence of a chaperone relay; inhibition of HSP90 trapped the receptor in a complex with heat-shock protein 70 (HSP70) and additional co-chaperones.

## EXPERIMENTAL PROCEDURES

### 

#### 

##### Receptor Constructs

**FIGURE 1.**
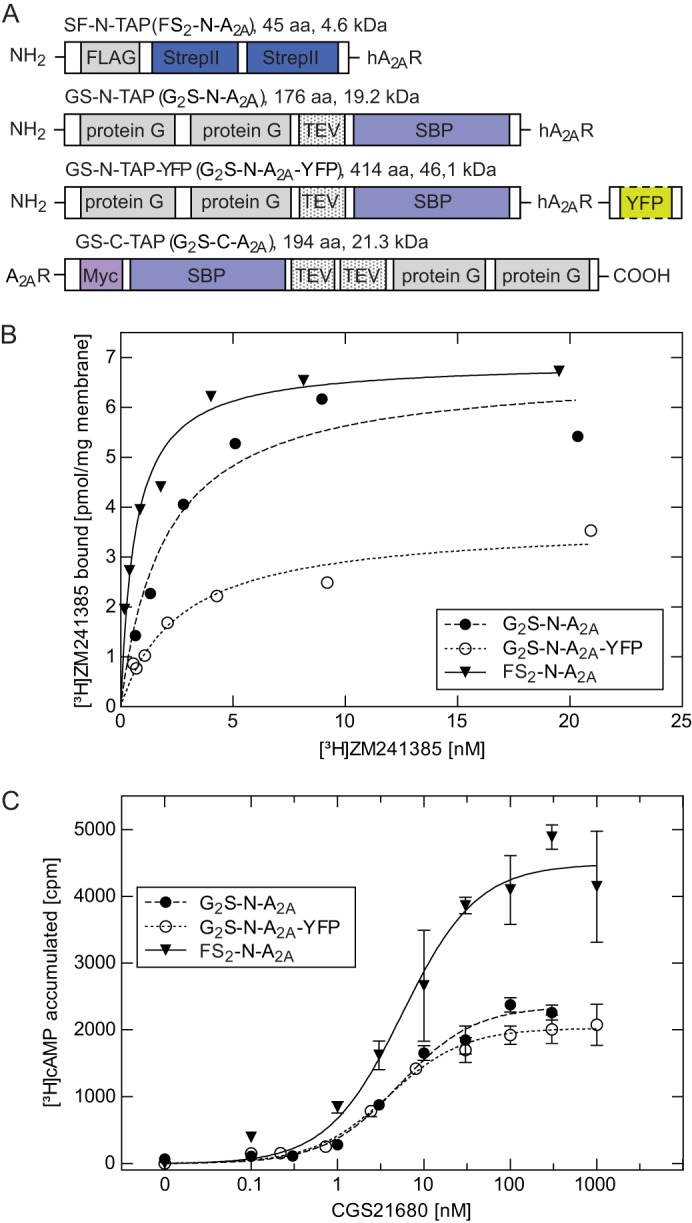
**Expression of N-terminally tagged A_2A_ receptor constructs in HEK293 cells.** *A*, schematic representation of the differently tagged receptors employed; G_2_S-N-TAP (total size 176 amino acids, 19.2 kDa) and the G_2_S-C-TAP (194 amino acids, 21.3 kDa) contain two protein G moieties and a streptavidin binding protein (*SBP*) fused to the N terminus and C terminus, respectively. G_2_S-N-TAP-A_2A_R-YFP contains, in addition, a YFP moiety at the C terminus. FS_2_-N-TAP (45 amino acids, 4.6 kDa) contains a FLAG-epitope and two Strep-Tactin moieties at the N terminus of the receptor. *B*, membranes (5–10 μg/assay) prepared from HEK293 cells stably expressing the indicated receptor (G_2_S-N- A_2A_R, G_2_S-N-TAP-A_2A_R-YFP, and FS_2_-N-A_2A_R) were incubated with increasing concentrations of the antagonist radioligand [^3^H]ZM241385. Data are means from duplicate determinations in a representative experiment. Two additional clones per construct were characterized. *C*, HEK293 cells (4 × 10^5^) stably expressing the indicated receptor (G_2_S-N-A_2A_R, G_2_S-N-TAP-A_2A_R-YFP, and FS_2_-N-A_2A_R) were incubated with [^3^H]adenine to metabolically label their adenine nucleotide pool. Accumulation of [^3^H]cAMP was measured after stimulation with the indicated concentrations of CGS21680. Data are means ± S.D. (*error bars*) from three independent experiments. One representative clone per construct is shown.

The cDNA encoding the human A_2A_ receptor was amplified with the Finnzyme Phusion^TM^ high fidelity polymerase system (Thermo Scientific, Waltham, MA) using primers that introduced FseI and NotI restriction sites at the 5′- and 3′-ends, respectively. The resulting PCR product was subcloned into the pCeMM-GS-N-TAP plasmid (16) using restriction endonucleases (New England Biolabs (Ipswich, MA)/Fermentas-Thermo Scientific) and T4 DNA ligase (Fermentas-Thermo Scientific). The resulting construct is referred to as the G_2_S-N-A_2A_ receptor. The G_2_S-N-A_2A_ receptor construct was digested with EcoRI and NotI endonucleases and ligated into a pCDNA3 expression plasmid (Invitrogen). The C-TAP construct was generated in a similar manner using FseI/EcoRI restriction sites and subcloning into the pCeMM GS-C-TAP (17). Both plasmids contained two protein G moieties, a tobacco etch virus (TEV) cleavage site and a streptavidin-binding protein. GS-N-TAP-YFP (hereafter referred to as the G_2_S-N-A_2A_-YFP receptor) construct was cloned in a similar manner using an A_2A_-YFP (C-terminal) plasmid as a PCR template. As an alternative affinity tag, a combination of a tandem Strep-Tactin tag II and a FLAG tag (SF-TAP) replaced the protein G/streptavidin-binding protein sequence, resulting in the SF-N-TAP receptor construct (hereafter referred to as the FSt_2_-N-A_2A_ receptor); the A_2A_ receptor cDNA was amplified to contain NheI and ApaI restriction sites and cloned into pCDNA/SF-NTAP plasmid for expression (16). This yielded the construct with N-terminal fusion of a single FLAG epitope and a StrepII (Strep-Tactin) affinity moiety. All receptor constructs are schematically illustrated in Fig. 1*A*. Each expression plasmid was verified by DNA sequencing prior to transfection.

##### Cell Culture, Cell Transfection, and Membrane Preparation

Stably transfected cell lines were generated using the expression plasmids outlined above as described previously (3). Mock-transfected and stably transfected cell lines expressing the tagged A_2A_ receptors were kept in culture media containing Dulbecco's modified Eagle's medium (DMEM; PAA Laboratories (Pasching, Austria)) and 10% fetal calf serum (FCS; Invitrogen) at 37 °C. The amount of CO_2_ was 5%. PC12 cells were propagated as described previously (18).

HEK293 cells stably expressing the FS_2_-N-A_2A_ receptor were transfected with 30 nm Silencer® Select predesigned siRNAs (s6993 and s6994 for HSP90α; s21074 and s21075 for P23; s195025 and s195026 for CHIP) or Silencer® Select negative control siRNA (Ambion, Invitrogen) using Lipofectamine® RNAiMAX reagent. After 48 h, cell lysates were prepared for immunoblotting with antibodies against HSP90α, P23, CHIP (Abcam), or the A_2A_ receptor (Merck-Millipore). The levels of G protein β-subunits were quantified as a loading control with antiserum 7, which recognizes residues 8–23 in the G protein β1- and β2-subunits (19). Cells from parallel transfections were assayed for whole cell radioligand binding (see below). Cell membranes were prepared according to Klinger *et al.* (3).

##### Accumulation of cAMP

Stable cell lines were grown in poly-d-lysine (Merck-Millipore)-coated 6-well plates. The adenine nucleotide pool was metabolically labeled by incubating confluent monolayers for 16 h with [^3^H]adenine (1 μCi/well, PerkinElmer Life Sciences) as described (3). After the preincubation, fresh medium was added that contained 100 μm Ro-20-1724 (a phosphodiesterase inhibitor; Calbiochem-Merck Millipore) and adenosine deaminase (2 units/ml; Roche Applied Science) to remove any endogenously produced adenosine. After 4 h, cAMP formation via receptor was stimulated by the A_2A_-selective agonist CGS21680 (1 nm to 10 μm; Sigma-Aldrich) or directly by 30 μm forskolin (Sigma-Aldrich) for 20 min at 37 °C. Each experiment was performed in triplicate.

##### Radioligand Binding Assays

Membranes (25–100 μg/assay) from PC12 cells or HEK293 cells stably expressing the tagged A_2A_ adenosine receptors were incubated in a final volume of 0.2 ml containing 50 mm Tris-HCl (pH 8.0), 1 mm EDTA, 5 mm MgCl_2_, 8 μg/ml adenosine desaminase, and logarithmically spaced concentrations (0.5–25 nm) of [^3^H]ZM241385 (American Radiolabeled Chemicals, St. Louis, MO). After 60 min at 23 °C, the reaction was terminated by rapid filtration over glass fiber filters (Whatman-GE Healthcare). Nonspecific binding was determined in the presence of 5–10 μm xanthine amine congener (XAC; Sigma-Aldrich) and represented about 10% of total binding at 2 nm [^3^H]ZM241385. Specific binding represents the difference between total and nonspecific binding. Incubations were considered to represent binding to intact cells only if >90% of the cells became adherent upon replating after a mock incubation. Binding to intact cells was monitored as described (7) with the following modifications. In brief, HEK293 cells stably expressing the NTAP-A_2A_ receptor (1.6 × 10^5^ cells) were incubated in medium (DMEM containing 0.5% FCS and 5 μg/ml adenosine deaminase) at a final concentration of 2 nm [^3^H]ZM241385 for 15 min at 23 °C. Nonspecific binding was defined by the addition of CGS21680 (100 μm) or XAC (10 μm). The reaction was terminated by rapid filtration over glass fiber filters (Whatman-GE Healthcare). Assays were done in quadruplicate. Intracellular, binding-competent receptors were also quantified in PC12 cells (3.5 × 10^5^ cells/assay) and HEK293 cells stably expressing N-tagged A_2A_ receptor (2 × 10^5^ cells/assay) that had been pretreated for 24 h in the presence of the HSP90 inhibitors radicicol (Sigma-Aldrich) and 17-dimethylaminoethylamino-17-demethoxygeldanamycin (17-DMAG; Sigma-Aldrich). Surface receptors were quantified by measuring the difference before and after an acid strip (50 mm glycine, 125 mm NaCl, pH 3.0) (21). Total receptor numbers were also determined by measuring the radioactivity released after dissolving the samples in 1 m NaOH (22). Parallel incubations were done in the presence of 10 μm XAC to define nonspecific binding. The number of viable cells was determined manually using a microscope counting chamber.

##### Epifluorescence Microscopy and Imaging of N-terminally Tagged A_2A_ Receptor

HEK293 cells stably expressing the G_2_S-N-A_2A_-YFP receptor were seeded on PDL-covered glass coverslips into 6-well tissue culture dishes and allowed to adhere for ≥4 h. Thereafter, the coverslips were transferred into a microscopy chamber and overlaid with Krebs-HEPES buffer. Receptor distribution was visualized by epifluorescence microscopy (Zeiss Axiovert 200).

##### Purification of N-terminally Tagged A_2A_ Receptor

Receptors were solubilized with the non-ionic detergent *n*-dodecyl-β-d-maltoside (DDM; Thermo Scientific) in solubilization buffer (25 mm HEPES, pH 7.5), containing protease inhibitor mixture (Roche Applied Science). The optimum concentration of DDM was found to be 0.1–0.5% using a ratio of detergent to membrane protein of 4:1–8:1 (w/w). Samples were incubated at 4 °C for 30 min on a rotating wheel, followed by gentle homogenization with a hypodermic needle (0.4 × 20 mm; Henry Schein (Gillingham, UK)). Insoluble and solubilized material was separated by centrifugation at 100,000 × *g* for 1 h. Prior to TAP purification, the DDM concentration was diluted to 0.1–0.2%. The solubilized G_2_S-N-tagged receptor was purified as described earlier (17) with minor modifications. Briefly, the tagged receptor was incubated with IgG-agarose (Sigma-Aldrich) for 2 h at 4 °C to afford enrichment via the protein G moiety in the TAP tag. The protein complexes were eluted from the beads by cleavage (on column) using TEV protease (Promega) for 1 h at 16 °C. The eluted protein complex was then incubated with streptavidin-conjugated beads (Thermo Scientific), which selectively bind the SBP binding peptide of the TAP tag, for 2 h at 4 °C. Finally, the purified complex was eluted from the beads by incubation with 2 mm biotin in buffer containing 100 mm Tris-HCl, pH 7.8, and 6 m urea (both Sigma-Aldrich) and used for in-solution digest and MS analysis. Solubilization of FSt_2_-N-A_2A_ was performed in the same manner. TAP was performed as described earlier (16). Alternatively, single step affinity purifications using either affinity tags were carried out to reduce the amount of starting material needed. All solubilization and purification procedures were simultaneously performed with untransfected HEK293 cells to account for false negative MS results. Purification was monitored by blotting for the receptor using Streptavidin HRP-conjugated binding protein (Thermo Scientific), anti-FLAG antibody (Sigma-Aldrich), or an anti-A_2A_ receptor-specific antibody (Merck-Millipore). The bound detection agent was visualized by enhanced chemiluminescence with Pico or Femto reagent (Thermo Scientific). The chemiluminescence was captured with the FluoChem HD2 CCD camera (Biozym; Hessisch Oldendorf, Germany) and quantified with ImageJ software using the gel analysis feature. The FLAG-BAP^TM^ fusion protein (Sigma-Aldrich) was used as a reference standard.

##### Tryptic Digest, Nano-LC-MS/MS, and Protein Database Analysis

Eluted TAP proteins were digested in solution. Samples were denatured in 100 mm Tris-HCl, pH 7.8, 6 m urea for 10 min at 65 °C, followed by reduction (using 200 mm DTT; Sigma-Aldrich) for 1 h at 23 °C and alkylation (using 400 mm iodoacetamide; Sigma-Aldrich) for 1 h at 23 °C. Proteins were desalted and concentrated prior digest by centrifugation in YM3 spin columns (Merck-Millipore) for 45 min at 4 °C. Proteins were digested with an excess of trypsin (MS grade; Sigma-Aldrich) for 16–18 h at 37 °C. Digest was quenched by adding 10% formic acid and analyzed by nano-LC-MS/MS. A Dionex Ultimate 3000 nano-HPLC system (Dionex-Thermo Scientific) was used for reversed phase separation on a Dionex Acclaim PepMap C_18_ 100-Å column (150 mm × 75 μm; particle size = 3 μm) prior to MS. The HPLC system was operated in preconcentration mode using 0.1% aqueous trifluoroacetic acid as a loading solvent. An aliquot of the sample (1–5 μl) was injected onto the HPLC column. The mobile phase consisted of solvent A (0.1% aqueous formic acid) and solvent B (80:20 (v/v) acetonitrile, 0.08% aqueous formic acid). Tryptic peptides were eluted using a gradient elution program of 4–60% B in 100 min, 60–90% B in 1 min, and finally a 5-min hold at 90% B, followed by a return to 4% B for a 10 min equilibration. The flow rate was 300 nl/min. The eluate from the RP-HPLC column was directly introduced into the nanospray source. Mass spectrometry was done on a hybrid quadrupole/linear ion trap 4000 QTRAP MS/MS system (ABSciex; Framingham, MA). The 4000 QTRAP equipped with a nanospray source was operated in positive ionization mode. All analyses were performed using information-dependent acquisition and the linear ion trap acquisition modes. Analyst version 1.5 software was used for data analysis. The acquisition protocol used to provide mass spectral data for database searching involved the following procedure. Mass profiling of the HPLC eluant was performed using enhanced multiple scan, and ions over the background threshold were subjected to examination using the enhanced resolution scan to confirm charge states of the multiply charged molecular ions. The most and next most abundant ions in each of these scans with a charge state of +2 to +5 or with unknown charge were subjected to collision-induced dissociation using rolling collision energy. An enhanced product ion scan was used to collate fragment ions and present the product ion spectrum for subsequent database searches. Database searching of LC-MS/MS data were carried out using ProteinPilot^TM^ software (version 4.0) and the Paragon algorithm (ABSciex).

##### Co-affinity Precipitation of A_2A_ Receptor and Heat-Shock Proteins

HEK293 cell lines stably expressing N-tagged A_2A_ receptor or PC12 cells were propagated in 15-cm dishes and treated with 17-DMAG or radicicol (0.1–10 μm), 2 μm kifunensine, or 10 nm bortezomib for 18–24 h in 10-cm dishes. Cells (1–5 × 10^7^ cells) were harvested, and membranes were prepared as described previously (14). Membranes were solubilized as described above, followed by incubation with streptavidin-conjugated beads (G_2_S-N-A_2A_) or Strep-Tactin-Sepharose beads (FSt_2_-N-A_2A_) for 2 h at 4 °C. Beads were washed twice with solubilization buffer; the bound protein was released by the addition of 0.1 ml of sample buffer and an incubation at 45 °C for 25 min. After centrifugation for 3 min at 13,000 × *g*, the supernatant was collected and separated by SDS-polyacrylamide gel electrophoresis. The endogenously expressed A_2A_ receptor of PC12 cells was immunoprecipitated with the anti-A_2A_ receptor antibody; membranes (1 mg) were extracted, and the solubilized supernatant containing the solubilized A_2A_ receptor was mixed with anti-A_2A_ receptor antibody (12.75 μg) and incubated for 16 h at 4 °C. Thereafter, preequilibrated protein G-Sepharose beads were added and incubated for an additional 3 h at 4 °C. The beads were washed twice with 0.5 ml of solubilization buffer. Bound proteins were eluted by the addition of reducing sample buffer and an incubation at 45 °C for 25 min. A_2A_ receptor and HSP90 were detected by immunoblotting using either anti-A_2A_R or anti-FLAG and anti-HSP90α (Abcam, Cambridge, UK) antibodies, respectively. As a control, membranes were harvested from untransfected HEK293 cells and processed in parallel. Immunoblots were quantified as described above. Where required, nitrocellulose membranes of primary and secondary antibody labeling were stripped by incubation with KSCN buffer (3 m KSCN, 150 mm KCl, 10 mm NaH_2_PO_4_, 100 mg/liter polyvinylpyrrolidone K30, 1-ethenylpyrrolidin-2-one) for 20 min at 23 °C to afford detection of additional proteins with the pertinent antibodies (*i.e.* anti-HSP90β, anti-HSC70, anti-HOP, anti-P23, and anti-CHIP, all from Abcam).

##### Enzymatic Deglycosylation

HEK293 cells (1–5 × 10^7^) stably expressing the tagged receptor were rinsed with ice-cold PBS, detached with a cell scraper, and harvested by centrifugation at 400 × g for 5 min. The pellets were washed once with ice-cold PBS, centrifuged, and resuspended in buffer (20 mm Hepes-NaOH, pH 7.4, 1 mm EDTA, 2 mm MgCl_2_ containing protease inhibitors) and 1% Triton X-100 (Sigma-Aldrich). The solubilized proteins were deglycosylated using either 2500 units of endoglycosidase H or 500 units of peptide:*N*-glycosidase F according to the protocol provided by the manufacturer (New England Biolabs). Briefly, after denaturation in 5% SDS and 0.4 m DTT, the reaction was done in 50 mm sodium citrate (pH 5.5) for endoglycosidase H or 50 mm sodium phosphate (pH 7.5) containing 1% Nonidet P-40 for peptide:*N*-glycosidase F, respectively, for 16 h at 37 °C. Reaction products were visualized by immunoblotting using the antibody directed against the A_2A_ receptor.

##### MBP Pull-down Assays

MBP-A_2A_-C-tail (a fusion protein comprising maltose-binding protein and the C terminus of the A_2A_ receptor and MBP) was purified from *Escherichia coli* lysates, and pull-down assays were done as described (9) with the following modifications. MBP-A_2A_-C-tail or MBP (15 μg each) fusion protein was incubated with recombinant HSP90 (1 μg; Enzo Life Sciences, Farmingdale, NY) in a total volume of 45 μl. The incubation lasted for 2 h at 30 °C, and the proteins were recovered with amylose (50 μl of a preequilibrated 1:1 slurry).

## RESULTS

### 

#### 

##### Characterization of A_2A_ Receptor Constructs

The power of co-affinity purification has been enhanced by the strategy of TAP (16, 17). The available tags were originally designed for the purification of soluble, cytoplasmic proteins (16, 17). Accordingly, we examined several constructs (see *schematic representation* in Fig. 1*A*); when stably expressed in HEK293 cells, both N- and C-terminally tagged constructs gave rise to receptors that bound the antagonist with an affinity indistinguishable from the wild type A_2A_ receptor (Fig. 1*B*). However, in cells expressing the C-terminally tagged receptor, the concentration-response curve for agonist-induced cAMP accumulation was shifted to the right by about 20-fold, when compared with N-terminally tagged and untagged receptors (Fig. 1*C*). This shift was seen in three independently generated clonal cell lines (Table 1). Hence, we concluded that the C-terminal addition of two protein G moieties and an SBP moiety interfered with coupling to G_s_. Accordingly, this construct was not used for TAP. In contrast, the FSt_2_-N-A_2A_ receptor and the G_2_S-A_2A_ receptor were functionally indistinguishable from the wild type A_2A_ receptor. The majority of the subsequent experiments were performed with both constructs, and these gave equivalent results.

**TABLE 1:** **Binding and functional characteristics of TAP-A_2A_R receptors in HEK293 cells** Results from cAMP assays were fitted to the equation describing a hyperbola with a constant basal term. *E*_max_ refers to the maximum [^3^H]cAMP accumulation (cpm). Dissociation constants (*K_D_*) and the amount of A_2A_ receptors in the selected stably transformed cell clones were calculated by fitting specific binding of the antagonist [^3^H]ZM241385 to the equation for a rectangular hyperbola.

Construct	EC_50_	*E*_max_	*K_D_*	*B*_max_
	*nm*	*cpm*	*nm*	*pmol/mg*
G_2_S-N-hA_2A_R-4	5.2 ± 1.2	2354 ± 75	2.0 ± 0.7	6.8 ± 0.7
G_2_S-N-hA_2A_R-8	3.6 ± 1.6	958 ± 92	2.6 ± 0.5	3.0 ± 0.2
G_2_S-N-hA_2A_R-9	5.1 ± 1.8	1804 ± 221	2.1 ± 0.3	2.5 ± 0.01
G_2_S-C-hA_2A_R-1	109 ± 1.6	2623 ± 346	4.2 ± 1.1	0.9 ± 0.1
G_2_S-C-hA_2A_R-5	170 ± 1.4	3504 ± 358	4.5 ± 2.0	1.4 ± 0.2
G_2_S-C-hA_2A_R-6	140 ± 1.5	2276 ± 273	4.0 ± 0.8	1.0 ± 0.1
FS_2_-N-hA_2A_R-5	2.9 ± 1.6	3358 ± 251	2.6 ± 1.1	14.5 ± 2.0
FS_2_-N-hA_2A_R-7	5.6 ± 1.1	4484 ± 166	1.3 ± 0.1	7.0 ± 0.3
FS_2_-N-hA_2A_R-10	6.8 ± 1.6	3182 ± 219	2.4 ± 0.9	11.5 ± 1.3

**FIGURE 2.**
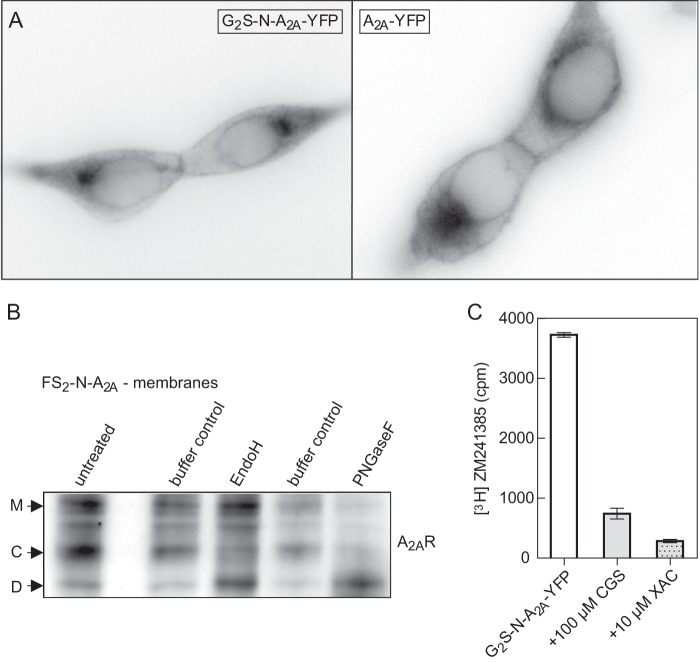
**Localization of tagged A_2A_ receptors in HEK293 cells.** *A*, epifluorescence microscopy images of G_2_S-N-A_2A_R-YFP fusion protein (*left*) and A_2A_R-YFP (*right*), transiently expressed in HEK293 cells. *B*, representative deglycosylation experiment showing differently glycosylated species of the A_2A_ receptor: mature (*M*), core-glycosylated (*C*), and deglycosylated (*D*). Volume-corrected aliquots (*i.e.* corresponding to 3 × 10^5^ cells) of HEK293 cell membranes stably expressing FS_2_-N-A_2A_R (*n* = 3) were incubated with either 2500 units of endoglycosidase H (*EndoH*; *lane 3*), 500 units of peptide:*N*-glycosidase F (*PNGaseF*; *lane 5*), or assay buffers only (*lanes 2* and *4*). Different A_2A_R glycoforms were compared with untreated cell membranes (*lane 1*). *C*, representative experiment to determine the location of ligand binding-competent A_2A_ receptor. HEK293 cells stably expressing G_2_S-N-A_2A_R-YFP (2 × 10^5^ cells) were incubated with a 2 nm concentration of the membrane-permeable antagonist [^3^H]ZM241385 in the absence (*white bar*) and presence of the hydrophilic agonist CGS21680 (100 μm; *gray bar*) or the membrane-permeable antagonist XAC (10 μm; *dotted bar*) alone. *Error bars*, S.D.

As noted previously (9, 14, 15), substantial amounts of the A_2A_ receptor are retained in the ER. Accordingly, we visualized the cellular distribution of the N-terminally tagged receptor in stably transfected cells (Fig. 2*A*). We confirmed that the fraction of the receptor that was retained within the cell had only undergone core glycosylation by determining its susceptibility to endoglycosidases; after electrophoretic separation, several immunoreactive bands were visualized (Fig. 2*B*, *left lane*, *bands* labeled *M*, *C*, and *D*). Control incubations with reaction buffer resulted in loss of immunoreactivity but did not change the relative abundance of these bands (Fig. 2*B*, *lanes* labeled *buffer control*). This presumably reflects proteolysis during the incubation. The addition of endoglycosidase H increased the intensity of the band denoted D at the expense of band C but did not affect the upper band M (Fig. 1*B*, *lane* labeled *EndoH*). In contrast, incubation in the presence of peptide:*N*-glycosidase F increased band D at the expense of both bands (Fig. 1*B*, *right lane*). Accordingly, we concluded that the endoglycosidase H-resistant band M represented the mature, fully glycosylated receptor, whereas the band C corresponded to the ER-retained core glycosylated receptor. Band D was the deglycosylated protein.

We surmised that the A_2A_ receptor that accumulated in the ER was retained because it had not attained its stably folded (*i.e.* binding-competent) conformation. We explored this conjecture by using the hydrophobic radioligand antagonist [^3^H]ZM241385 (log D at pH 7.4 = 2.82) and two unlabeled receptor ligands to define nonspecific binding; CGS21680 is a hydrophilic agonist that carries a ribose moiety and hence does not readily enter cells (log D at pH 7.4 = −2.17). In contrast, the antagonist XAC ought to permeate freely because of its amphipathic nature (log D at pH 7.4 = −0.04). As can be seen from Fig. 2*C*, an excess of CGS21680 displaces about 80% of [^3^H]ZM241385 bound to intact cells. Displacement by a saturating concentration of XAC was only slightly more effective. As an alternative approach, we determined the fraction of [^3^H]ZM241385 binding to intact cells that was released by an acid strip (7). This gave equivalent results (not shown; see Figs. 5–7). Based on these observations, we conclude that the receptors, which bind ligands, are predominantly at the cell surface. The bulk of the A_2A_ receptor retained within the cell is not in a conformation conducive to antagonist binding.

##### TAP of N-terminally Tagged A_2A_ Receptor and Analysis of the Associated Protein by Mass Spectrometry

**FIGURE 3.**
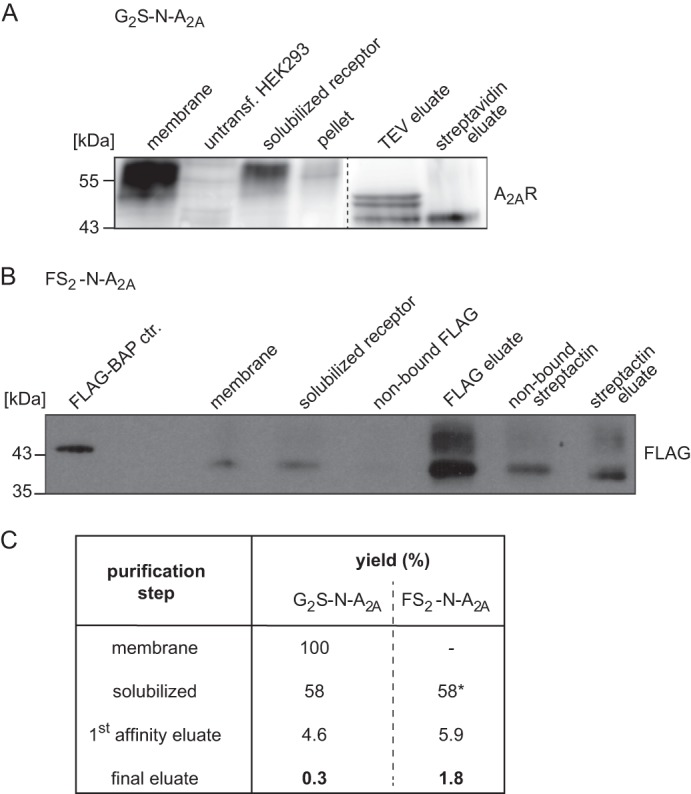
**Solubilization and purification of N-terminally tagged A_2A_ receptors.** *A*, purification of G_2_S-N-A_2A_R stably expressed in HEK293 cells (*lane 1*) was carried out in comparison with untransfected HEK293 cells (*lane 2*). They were monitored by Western blot (anti-A_2A_R) and CCD image analysis. The amount of solubilized G_2_S-N-A_2A_R is shown in *lane 3*, and the insoluble pellet is shown in *lane 4* (both in equivalent volumes corresponding to 25 μg of original membranes). *Lanes 5* and *6* represent the TEV eluate (equivalent to 10 times the original volume) and streptavidin eluate (equivalent to 100 times the original volume). *B*, aliquots at crucial steps of FS_2_-N-A_2A_R purification (corresponding to 32 μg of original membrane protein) (*lanes 2–7*, membranes, solubilized receptor, non-bound FLAG-agarose supernatant, FLAG eluate, non-bound Strep-Tactin supernatant, and final Strep-Tactin eluate, respectively) and 10 ng of FLAG-BAP control (*lane 1*) were quantified using scanned Western blot images (anti-FLAG). *C*, the yields of recovered protein complexes of typical N-tagged-A_2A_R purifications were calculated from pixel intensities of the respective bands, corrected for the volumes, and normalized to isolated membrane (100%, G_2_S-N) or solubilized aliquot (58%, FS_2_-N), respectively.

Because a large quantity of the retained A_2A_ receptor failed to bind antagonist, we assumed that part of it could be stalled in an unproductive but folding-competent state (23–25). We used the affinity tags to enrich the receptor from detergent extracts and to examine the proteins associated with the receptor in detergent extracts. DDM was the most effective detergent for receptor solubilization. For the purification procedure, ≥3 × 10^8^ HEK293 cells (30 mg of membrane protein) stably expressing the N-tagged A_2A_ receptor were used as starting material. The purification of the N-terminally tagged constructs involved the following steps: (i) a first affinity purification via binding to IgG- or anti-FLAG-agarose and elution by TEV protease-dependent cleavage or with the FLAG peptide, (ii) a second affinity purification with streptavidin-linked beads or Strep-Tactin-Sepharose, (iii) final elution from the beads, and (iv) in-solution digest followed by nano-LC-MS/MS analysis. A representative enrichment is documented in Fig. 3, *A* and *B*, for G_2_S-N-A_2A_ receptor and FSt_2_-N-A_2A_ receptor, respectively. Yields were ∼0.3 and 1.8% in the final eluate, respectively (Fig. 3*C*).

The final eluate was subjected to tryptic digestion and nano-LC-MS/MS. The analyzed peptides and peptide fragment sequences were searched against a database of all human proteins. This procedure of repeated purification and MS analysis of the two differently tagged A_2A_ receptors (FSt_2_-N and G_2_S-N) and extracts from HEK293 cells expressing an untagged A_2A_ receptor as a control to account for false positives. We identified the bait protein (*i.e.* the A_2A_ receptor with sequence coverage of 69%) (Table 2). As can be seen from Table 2, several proteins were co-purified with the A_2A_ receptor that are *bona fide* candidates in assisting its folding; the presence of some, such as calnexin (15) and protein-disulfide isomerase (26), is unremarkable, because they are known to engage essentially all membrane proteins on the lumenal side of the ER. However, the heat-shock proteins are of particular interest as potential A_2A_ receptor-interacting proteins, because their presence is predicted by the chaperone/COPII coat exchange model (4).

**TABLE 2:** **Folding interactome of TAP-hA_2A_R in HEK293 cells** Digested tandem affinity-purified human A_2A_ receptors were analyzed by nano-LC-MS/MS, and protein data were obtained from ProteinPilot^TM^ and Paragon algorithm analysis using the SwissProt human protein database.

Protein score	Sequence coverage	Accession number	Protein name	Significant peptides (>95%)	Molecular function
	**%**				
**Bait protein: TAP-tagged A_2A_ receptor**					
12.27	68.93	pir A48978	Adenosine A**_2A_** receptor, human	7	Receptor; GPCR

**Molecular chaperones**					
10.42	71.99	pir HHHU86 (spt P07900)	Heat-shock protein 90α	4	Chaperone
28.79	71.59	spt Q15084	Protein-disulfide isomerase A6 precursor (protein disulfide-isomerase P5)	16	Isomerase; chaperone
3.40	59.15	spt P14625	Endoplasmin precursor (94-kDa glucose-regulated protein; gp96 homolog, tumor rejection antigen 1)	2	Chaperone
30.56	82.73	spt P27797	Calreticulin precursor (CRP55) (calregulin, ERp60, grp60)	17	Calcium-binding protein
24.66	80.74	spt P27824	Calnexin precursor (major histocompatibility complex class I antigen- binding protein p88, p90, IP90)	16	Select calcium-binding protein; chaperone
20.19	92.82	trm Q5SP17	Heat-shock 70-kDa protein 1A (HSP70.1)	7	Chaperone

##### Co-affinity Precipitation of HSP90α and the A_2A_ Receptor

**FIGURE 4.**
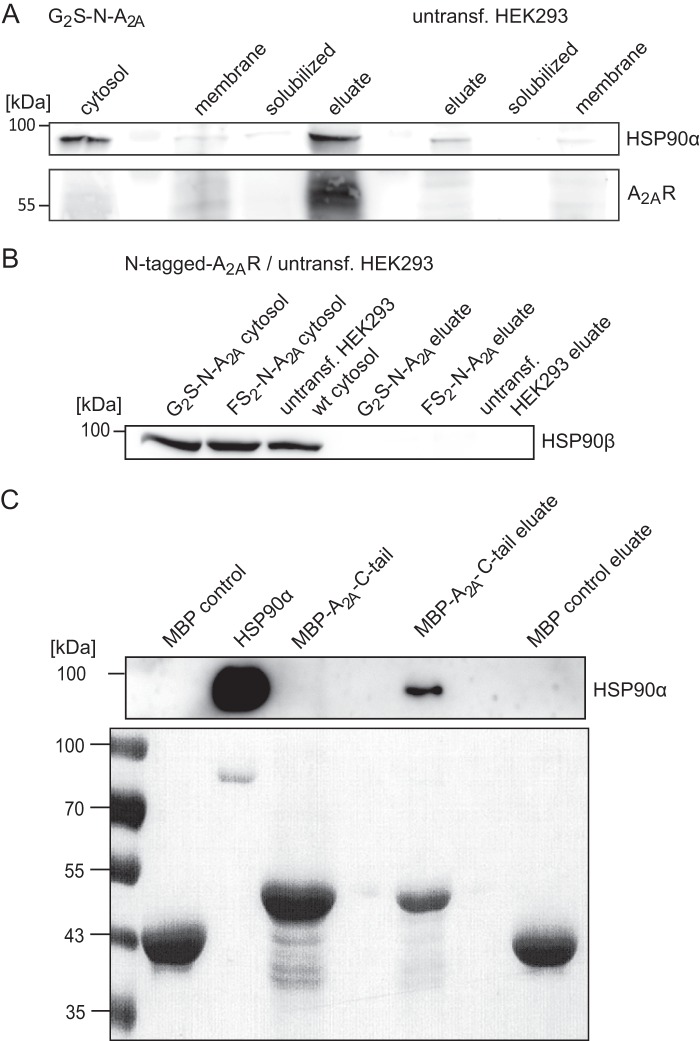
**Co-affinity precipitation of N-terminally tagged A_2A_ receptor and HSP90.** Membranes and a cytosolic fraction were prepared from cells stably expressing the tagged version of the A_2A_ receptor G_2_S-N-A_2A_R and from untransfected HEK293 cells. Membranes were solubilized with DDM, and the soluble extract was mixed with streptavidin beads. After three washes, bound proteins were released by heat denaturation in reducing sample buffer. Volume-corrected aliquots (*i.e.* corresponding to 2.5 × 10^5^ cells) of the cytosolic fraction, the membrane fraction, the solubilized material, and the final eluate were resolved by denaturing electrophoresis and immunoblotted for HSP90α (*A*, *top*), HSP90β (*B*), and the A_2A_ receptor (*bottom*; visualized with the anti-A_2A_ receptor). *C*, maltose-binding protein (MBP control) or MBP-A_2A_-C-tail fusion protein was incubated with recombinant HSP90α and bound to amylose resin. After two washes, bound proteins were released by heat denaturation in reducing sample buffer. Volume-corrected aliquots (50% of the original reaction) were immunoblotted for HSP90α (*C*, *top*). The proteins were also visualized by Ponceau staining of the nitrocellulose membrane to document the amount of MBP employed as input and recovered in the eluate (*C*, *bottom*).

We confirmed the association of HSP90α with the A_2A_ receptor by monitoring the co-enrichment of the proteins during affinity precipitation. As expected, HSP90α was predominantly found in the cytosolic fraction, and only minute amounts were found in the membranes or in detergent extracts thereof (Fig. 4*A*, compare *lanes 3* and *4*). However, if the N-terminally tagged A_2A_ receptor was purified from these extracts by binding to streptavidin-linked beads, HSP90α was also greatly enriched (Fig. 4*A*, *lane 5*). We verified that HSP90α was not enriched by nonspecific absorption to the affinity support by solubilizing membranes prepared from untransfected HEK293 cells and by exposing the resulting extract to the streptavidin-containing beads (Fig. 4*A*, compare the *three right-hand lanes*). As an additional control, we examined whether the second major cytoplasmic isoform HSP90β was also present in the co-affinity precipitate. HSP90β was found in abundant amounts in the cytosolic fraction of both untransfected HEK293 cells and cells expressing N-terminally tagged receptors, but it was not identified if complexes were analyzed by mass spectrometry (Table 1). Accordingly, HSP90β was not recovered in the co-affinity precipitate with the A_2A_ receptor (Fig. 4*B*). This provided further evidence for a specific interaction between the A_2A_ receptor and HSP90α. The intracellular loops of the A_2A_ receptor are short, but the C terminus is long. Hence, the candidate binding site is likely to reside in the C terminus. This is also predicted by the COPII-chaperone exchange model (5). Consistent with this conjecture, a fusion protein comprising the A_2A_ receptor C terminus and MBP (A_2A_R C-tail) bound purified HSP90α (Fig. 4*C*).

##### HSP90 Inhibitor-induced Changes in Surface Levels of the A_2A_ Receptor

**FIGURE 5.**
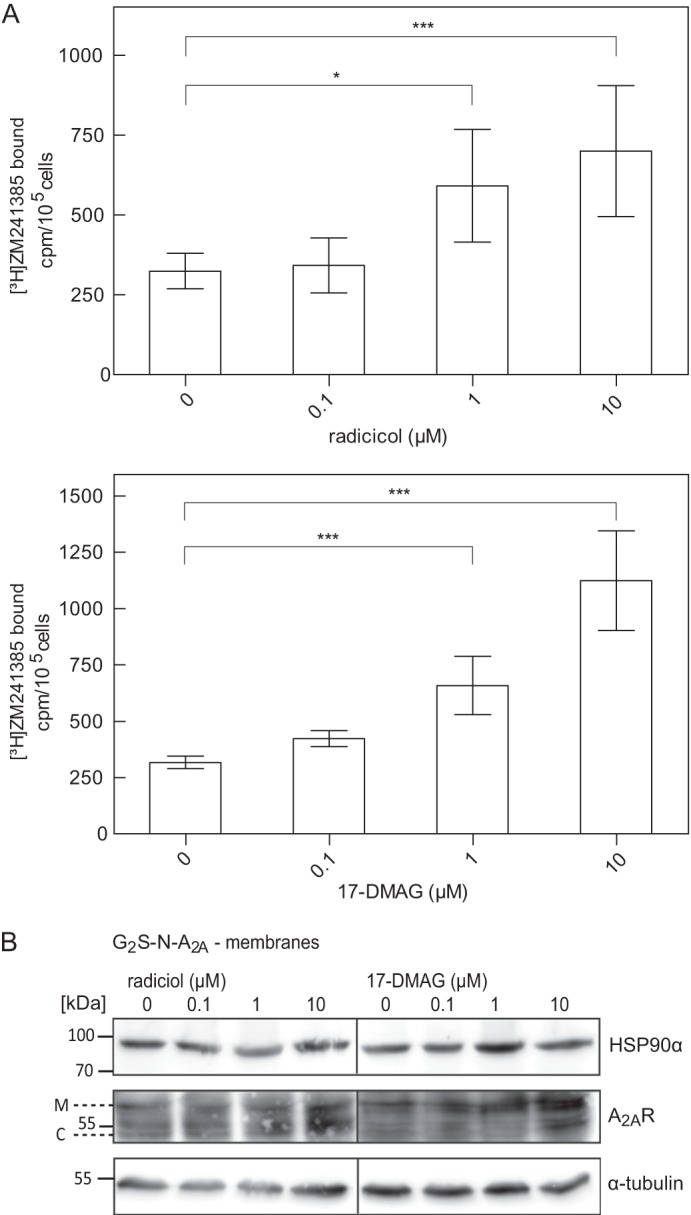
**Effect of HSP90 inhibitor treatment in total and surface levels of N-terminally tagged A_2A_ receptors.** *A*, HEK293 cells stably expressing the tagged receptor FS_2_-N-A_2A_R (2 × 10^5^ cells) were incubated with increasing concentrations of the HSP90 inhibitor radiciol or 17-DMAG for 24 h, followed by a 30-min incubation with the radioligand [^3^H]ZM241385 (1 nm) in the absence and presence of 10 μm XAC. After two washes, surface-bound antagonist was released by acid strip to determine the level of receptors at the cell surface. *Error bars*, S.D. Data are from four independent experiments done in triplicate. Statistical significance was calculated by analysis of variance followed by Dunnett's multiple-comparison post hoc test (*, *p* < 0.05; ***, *p* < 0.001). *B*, HEK293 cells stably expressing the tagged version G_2_S-N-A_2A_R were incubated with increasing concentrations of radicicol or 17-DMAG. After 24 h, cell membranes were prepared, and aliquots (corresponding to 2.5 × 10^5^ cells) were resolved by denaturing electrophoresis and immunoblotted for HSP90α (*top*), the A_2A_ receptor (*middle*), and α-tubulin (*bottom*; loading control).

**FIGURE 6.**
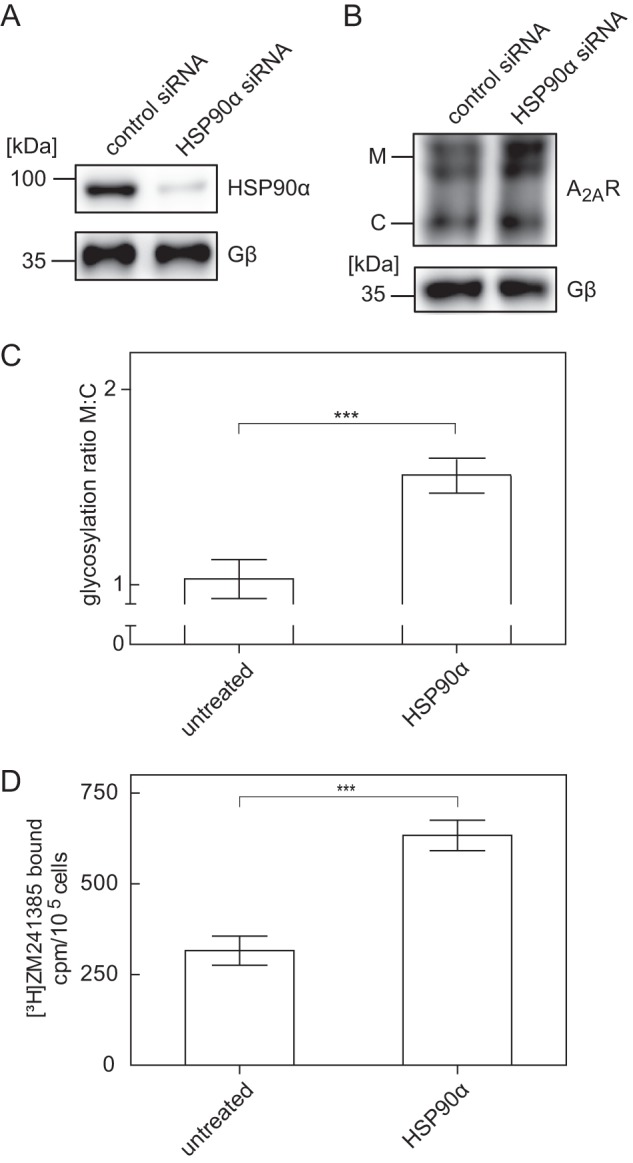
**Increase in total and surface levels of an N-terminally tagged A_2A_ receptor after siRNA-induced depletion of HSP90α.** *A*, HEK293 cells stably expressing the FS_2_-N-A_2A_ receptor were transfected with either negative control siRNA or siRNA targeting HSP90α. After 48 h, cells were lysed. Aliquots of the lysates were immunoblotted for HSP90α (*A*, *top*), A_2A_ receptor (*B*, *top*), or G protein β-subunits (*G*β, *lower blot* as loading control in *A* and *B*). *C*, immunoblots done as in *B*, *top*, were analyzed with ImageJ. The ratio of mature (*M*) and core (*C*) glycosylated species was determined and normalized by setting the mean ratio observed in untreated control cells as 1. Data are means from three independent experiments; *error bars*, S.D. Statistical significance was assessed with a paired *t* test (***, *p* < 0.001). *D*, HEK293 cells stably expressing the FS_2_-N-A_2A_ receptor (1.3 × 10^5^ cells) were transfected with either negative control siRNA or siRNA targeting HSP90α. After 48 h, cells were incubated with the radioligand [^3^H]ZM241385 (1 nm). Nonspecific binding was defined in the presence of 10 μm XAC. After two washes, surface-bound radioligand was released by acid strip and recovered in the supernatant. Data are means ± S.D. (*n* = 4); statistical significance was assessed with a paired *t* test (***, *p* < 0.001).

Our working hypothesis assumes that HSP90α binds to an ER-retained, stalled folding intermediate of the A_2A_ receptor. This may reflect overprotective quality control in the ER (9). Accordingly, we tested whether HSP90 inhibitors relaxed the stringency of quality control in the ER; cells were incubated in the presence of radicicol (Fig. 5*A*) or 17-DMAG (Fig. 5*B*) for 24 h, and cell surface levels of the A_2A_ receptor were assessed by measuring binding of [^3^H]ZM241385 that was sensitive to an acid strip. Both radicicol (Fig. 5*A*) and 17-DMAG (Fig. 5*B*) increased cell surface levels of the receptor in a concentration-dependent manner with EC_50_ values in the range of 400 nm and 1 μm, respectively. This is about 3-fold higher than their affinity for binding to HSP90 (27, 28), which suggests that a substantial fraction of the cellular HSP90 pool (*i.e.* >75%) must be inhibited before an effect on the A_2A_ receptor becomes detectable. The alternative explanation is extensive degradation of the HSP90 inhibitors. This appears unlikely, because 17-DMAG is very stable, with a half-life of 24 h, when administered to people (29). In fact, the potency of the compounds was not enhanced if the medium was replaced with fresh medium every 6 h. Similar observations were made with geldanamycin and by assessing cell surface receptors using displacement of the radioligand with the membrane-impermeable agonist CGS21680 (data not shown). The binding experiments suggested that the mature, fully glycosylated form of the receptor ought to be more abundant after incubation of cells in the presence of the HSP90 inhibitors. This was the case (Fig. 5*C*). However, saturating concentrations of both radiciol and 17-DMAG also resulted in increased accumulation of the core glycosylated band (Fig. 5*C*). Finally, we depleted HSP90α by siRNA-induced down-regulation (Fig. 6*A*). This manipulation also augmented the levels of the mature form of the A_2A_ receptor (Fig. 6, *B* and *C*). Accordingly, there were more binding-competent receptors at the cell surface (Fig. 6*D*).

##### Evidence for an HSP Relay in the Maturation of the A_2A_ Receptor

**FIGURE 7.**
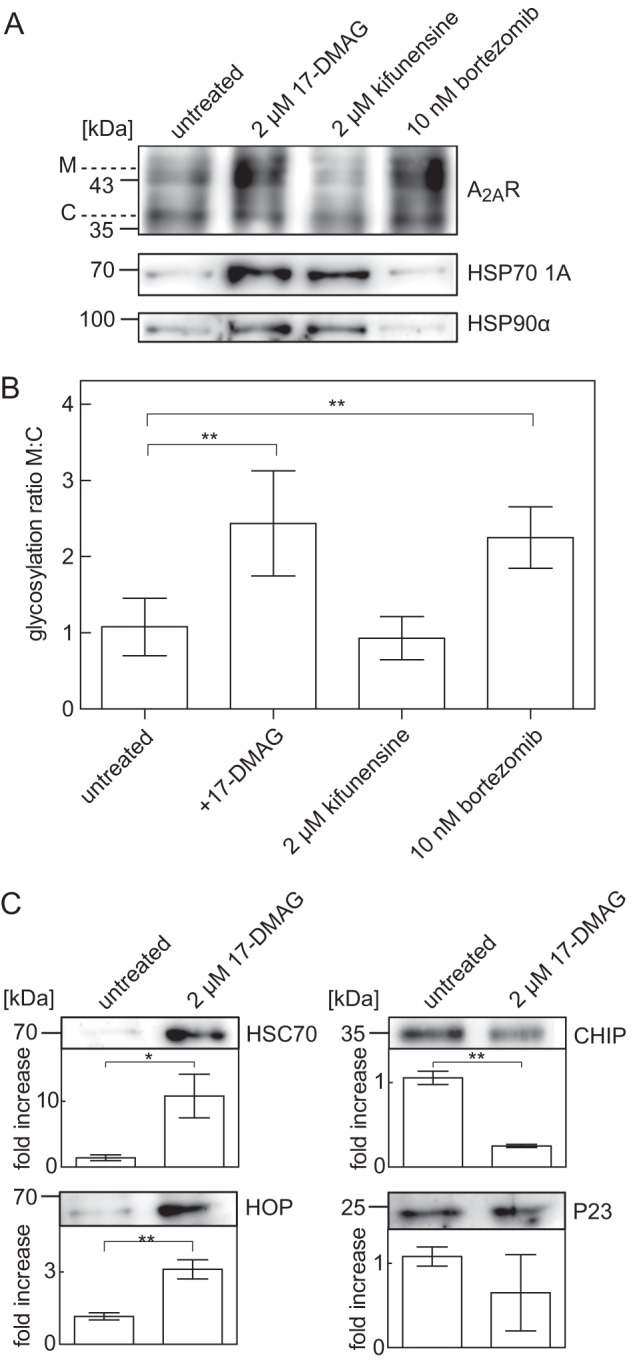
**Formation of receptor-chaperone complexes in the absence and presence of inhibitors of HSP90, mannosidase, and the proteasome.** *A*, HEK293 cells stably expressing the tagged receptor FS_2_-N-A_2A_R were incubated for 24 h in the absence (untreated; *lane 1*) or presence of 17-DMAG (2 μm; *lane 2*), kifunensine (2 μm, *lane 3*), or bortezomib (10 nm; *lane 4*). After membrane solubilization and immunoprecipitation, volume-corrected aliquots (*i.e.* corresponding to 2.5 × 10^5^ cells) of their co-affinity precipitation products were immunoblotted for A_2A_ receptor (*A*, *top*), HSP70-1A (*A*, *middle*), and HSP90α (*A*, *bottom*). *B*, immunoblots done as in *A* (*top*) were analyzed with ImageJ. The ratio of mature (*M*) and core (*C*) glycosylated species was determined and normalized by setting the mean ratio observed in untreated control cells as 1. Data are means from four independent experiments; *error bars*, S.D. Statistical significance was assessed by analysis of variance (**, *p* < 0.01). *C*, the A_2A_ receptor was immunoprecipitated from cells that had been incubated in the presence and absence of 17-DMAG (2 μm) as in *A*. The levels of HSC70 (*C*, *top*), HOP (*C*, *second* from *top*), P23 (*C*, *third* from *top*), and CHIP (*C*, *bottom*) were determined by immunoblotting and quantified with ImageJ. The pixel density of each band was determined and normalized by setting the mean density observed in untreated control cells as 1. Data are means from three independent experiments; *error bars*, S.D. Statistical significance was assessed by an unpaired *t* test (*, *p* < 0.05; **, *p* < 0.01).

Apart from HSP90α, HSP70–1A also co-purified with the A_2A_ receptor. The folding of cytosolic proteins is assisted by sequential recruitment of HSP (30, 31); J domain proteins (which are HSP40 members) work in conjunction with HSP70 members to engage an initially unfolded polypeptide segment and transfer this to HSP90 or alternatively, if folding cannot proceed, to the degradation machinery. We explored if an analogous relay also operated on the A_2A_ receptor. Because selective HSP70 inhibitors are not available, we employed manipulations that inhibited the exit points (*i.e.* 17-DMAG to prevent transfer to HSP90 and kifunensine to inhibit the mannosidase required to enter ER-associated degradation (32)). Bortezomib, a high affinity inhibitor of the proteasome, was used as a positive control, because blockage of the proteasome increases cell surface levels of the A_2A_ receptor (9). Treatment of cells with 17-DMAG increased the amount of HSP90 that was co-affinity-purified with the A_2A_ receptor (see Fig. 7*A*, *second lane*). This was to be expected, because 17-DMAG also binds to HSP90 in complex to its client protein and precludes its release (33). It is worth noting that treatment with 17-DMAG also augmented the association of HSP70 with the A_2A_ receptor (see Fig. 7*A*, *second lane*). Blockage of the other exit point by kifunensine also resulted in the accumulation of HSP70 and of HSP90 on the A_2A_ receptor (see Fig. 7*A*, *third lane*). In contrast, inhibition of the proteasome by bortezomib caused a pronounced increase in the mature form of the A_2A_ receptor but did not cause the accumulation of receptors in complex with either HSP70 or HSP90 (see Fig. 7*A*, *fourth lane*). After treatment with 17-DMAG and bortezomib, a significant increase of the higher glycosylated, mature form of the co-purified A_2A_ receptor was observed, together with an increase in total receptor protein levels (Fig. 7*B*). Treatment with kifunensine resulted in no significant increase of either form (Fig. 7*B*).

The chaperone relay is assisted by co-chaperones (30). Accordingly, we also blotted for co-precipitated co-chaperone protein levels after trapping the receptor-HSP90 complex by pretreating cells with 17-DMAG. This manipulation resulted in a substantial increase in the amount of co-precipitated HSC70 and HOP (HSC70/HSP90-organizing protein) but caused a decline in the levels of associated C terminus of HSP70-interacting protein (CHIP) (Fig. 7*C*). On average, there was also a decrease in associated P23 (HSP90 co-chaperone), but this did not reach statistical significance (Fig. 7*C*, *bottom right*).

**FIGURE 8.**
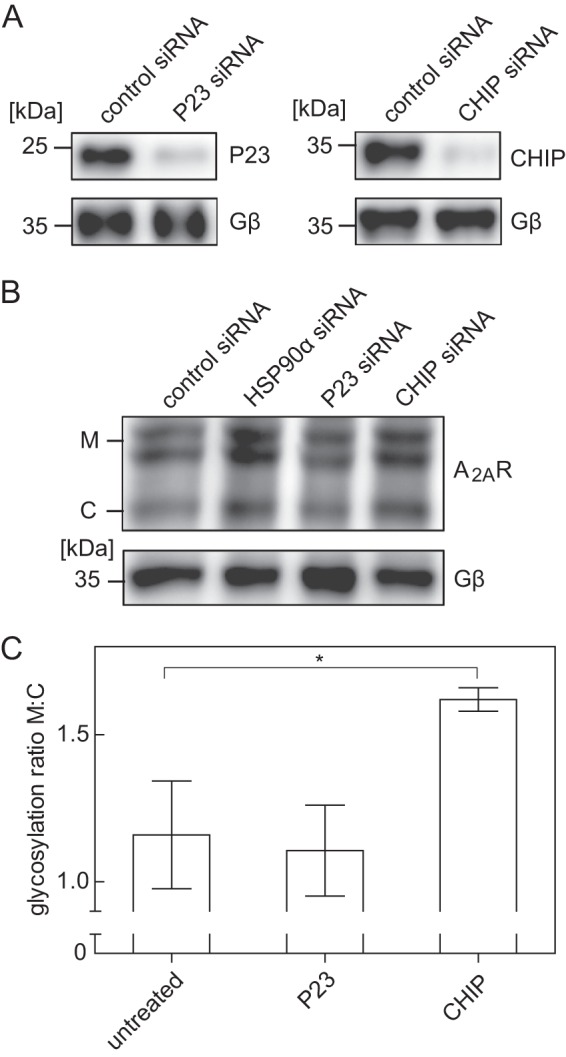
**Effect of siRNA-induced depletion of the co-chaperones P23 and CHIP on A_2A_ receptor levels.** *A* and *B*, HEK293 cells stably expressing the FS_2_-N-A_2A_ receptor were transfected either with negative control siRNA or with siRNAs targeting P23, CHIP, and HSP90α (as an internal reference, compare Fig. 6). After 48 h, cells were lysed. Aliquots of the lysates were immunoblotted for P23 (*A*, *top left*), CHIP (*A*, *top right*), and A_2A_ (*B*, *top*). G protein β-subunits (Gβ) were visualized as loading control (*lower blots* in *A* and *B*). *C*, immunoblots done as in *B* (*top*) were analyzed with ImageJ. The ratio of mature (*M*) and core (*C*) glycosylated species was determined and normalized by setting the mean ratio observed in untreated control cells as 1. Data are means from three independent experiments; *error bars*, S.D. Statistical significance was assessed by analysis of variance followed by Dunnett's multiple comparison post hoc test. *, *p* < 0.05.

If cellular CHIP levels were depleted by transfecting cells with the appropriate siRNAs (Fig. 8*A*, *right panel*), the mature form of the A_2A_ receptor increased to an extent that was comparable with that caused by depletion of HSP90α (Fig. 8*B*, compare *second* and *fourth lane*). In contrast, depletion of P23 (Fig. 8*A*, *left panel*) did not have any appreciable effect on A_2A_ receptor levels (Fig. 8, *B* (compare *first* and *third lane*) and *C*).

**FIGURE 9.**
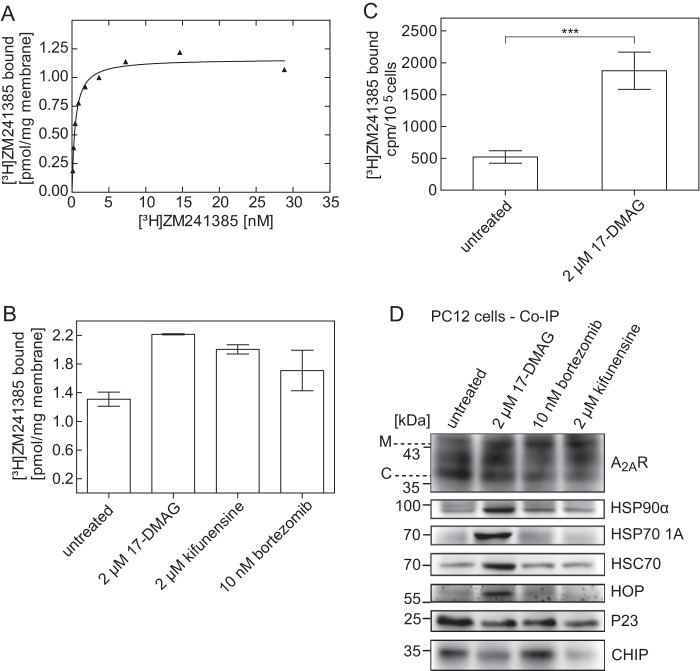
**Effect of HSP90 and/or proteasome inhibitors on the levels of endogenously expressed A_2A_ receptor in PC12 cells.** *A*, membranes (25–30 μg/assay) prepared from PC12 cells expressing the A_2A_ receptor were incubated in buffer with the indicated concentrations of [^3^H]ZM241385 in the absence and presence of XAC (10 μm) to define nonspecific binding. This nonspecific binding was <20% at 10 nm radioligand and subtracted to generate the saturation hyperbola. *B*, prior to membrane preparations of PC12 cells expressing the A_2A_ receptor, cells were incubated in the absence and presence of 17-DMAG (2 μm), kifunensine (2 μm), and bortezomib (10 nm). Membranes (25–30 μg/assay) were incubated with 10 nm [^3^H]ZM241385 in the absence and presence of 10 μm XAC to define nonspecific binding. *C*, PC12 cells endogenously expressing the A_2A_ receptor (3.5 × 10^5^ cells) were incubated with increasing concentrations of 17-DMAG, followed by incubation with the radioligand [^3^H]ZM241385 (5 nm). Nonspecific binding was defined in the presence of 10 μm XAC. After two washes, surface-bound radioligand was released by an acid strip and recovered in the supernatant. Assays were performed in quadruplicates. Data are means ± S.D. from four independent experiments; statistical significance was assessed with a paired *t* test (***, *p* < 0.001). *D*, PC12 cells endogenously expressing the A_2A_ receptor were incubated for 24 h in the absence (untreated; *lane 1*) or presence of 2 μm 17-DMAG (*lane 2*), 10 nm bortezomib (*lane 3*), or 2 μm kifunensine (*lane 4*). After membrane extraction and immunoprecipitation, aliquots (corresponding to 3 × 10^5^ cells) of the immunoprecipitate were blotted for the A_2A_ receptor (*top*), HSP90α, HSP70–1A, HOP, P23, and CHIP, as indicated. *M*, mature; *C*, core glycosylated.

We explored the association of A_2A_ receptors with HSP90α and HSP70-1A in PC12 cells to verify that the recruitment of a cytosolic HSP relay to the receptor did not simply result from its heterologous overexpression in HEK293 cells. PC12 cells express the A_2A_ receptor endogenously, but they are notorious for their genetic instability. This results in large variation in receptor levels (34). Accordingly, we determined the receptor levels in our cell population; the receptor density in a membrane preparation was about 1.2 pmol/mg (Fig. 7*A*). In PC12 cells, a large fraction of the A_2A_ receptor is retained within the cell (14); inhibition of the proteasome results in increased receptor levels (9). We pretreated the cells with 17-DMAG and kifunensine for 24 h and determined if this increased receptor levels by using a saturating concentration (10 nm; see Fig. 9*A*) of the radioligand [^3^H]ZM241385. As can be seen from Fig. 9*B*, preincubation with either 17-DMAG or kifunensine increased the levels of functional (*i.e.* binding-competent) receptors. In fact, the DMAG-induced increase in receptor density exceeds the effect elicited of the proteasome inhibitor bortezomib, which was used as a positive control. We verified that these additional receptors were at the cell surface as follows. (i) We determined the fraction of the radioligand that was released by an acid strip from intact cells (Fig. 9*C*). (ii) In addition, the receptor was immunoprecipitated, and the different species were resolved by denaturing electrophoresis; consistent with a large intracellular pool of A_2A_ receptors (14), the fully glycosylated, slowly migrating band represented only a fraction of the immunoreactive material (Fig. 9*D*, *left lane*, labeled *untreated*). Pretreatment with 17-DMAG, kifunensine, and bortezomib increased the abundance of this band at the expense of the most rapidly core-glycosylated band (Fig. 9*D*). HSP90α, HSP70–1A, and HSC70 were recovered in abundant amounts in the immunoprecipitate if the cells had been preincubated with 17-DMAG (Fig. 9*D*, compare *second* and *fourth blots*). Similarly, the levels of associated co-chaperones changed in a manner analogous to that seen in HEK293 cells (compare Fig. 7*C*); after pretreatment with DMAG, more HOP but less P23 and CHIP were recovered with the A_2A_ receptor (Fig. 9*D*, *last three blots*).

## DISCUSSION

The general machinery that assists the folding of membrane proteins on the lumenal side of the endoplasmic reticulum is understood in considerable detail (35, 36). In contrast, it is not clear how folding of GPCRs proceeds. There is only limited evidence that suggests that the folding trajectory is influenced by cytosolic components (reviewed in Ref. 37). The recently proposed chaperone-COPII exchange posits a crucial role of the C terminus as a binding site for the cytosolic folding machinery (5). The current experiments were designed to identify the nature of these cytosolic factors. The rationale for choosing the A_2A_ adenosine receptor is the observation that it incurs a folding problem (9, 15). The current findings support the conclusion that there is, in fact, a heat-shock protein relay system, which engages the A_2A_ receptor on the cytosolic side. This interpretation is supported by the following observations. (i) Two different TAP-tagged A_2A_ receptors afforded the purification of receptor-protein complexes in quantities that allowed for their analysis by mass spectrometry. This identified several molecular chaperones, in particular the heat-shock protein HSP90α and HSP701A. (ii) The presence of these chaperones was confirmed by both co-affinity precipitation from HEK293 cell extracts and co-immunoprecipitation from detergent-solubilized PC12 cell membranes. (iii) As predicted from its mechanism of action (32), the HSP90 inhibitor DMAG increased the amount of HSP90α that was recovered with the A_2A_ receptor. More importantly, pretreatment of cells with HSP90 inhibitors or siRNA-induced depletion of cellular HSP90α levels increased the cell surface level of the A_2A_ receptor in both HEK293 cells and PC12 cells. (iv) Co-chaperones were co-affinity-precipitated and co-immunoprecipitated with the A_2A_ receptor from detergent extracts of HEK293 cells and PC12 cells, respectively. Their association was modified upon HSP90 inhibition. The A_2A_ receptor is endogenously expressed in PC12 cells. The fact that essentially all observations were recapitulated in PC12 cells highlights the physiological relevance of the chaperone relay system and rules out the possibility that complex formation between heat-shock proteins and A_2A_ receptor is an artifact resulting from heterologous overexpression.

The cytosolic chaperones of the heat-shock protein family are thought to operate in a relay system; a representative of HSP40 (mammalian homologue of *E. coli* DnaJ, hence also referred to as J domain proteins) engages the unfolded peptide and delivers it to an HSP70, which is simultaneously converted to the ADP-bound state, which binds client peptides with high affinity (30). Subsequently, the partially folded client is transferred to an HSP90 (31). This transfer is typically assisted by HOP (Hsc70-Hsp90-organizing protein). Dimeric HS90 forms a clamp that promotes folding of the client. HSP90 is assisted by additional cofactors (*e.g.* P23, which stabilizes the dimer). Blockage of HSP90 resulted in the formation of stalled complexes that contained both HSP90 and HSP70/HSC70 and the transfer protein HOP. In contrast, P23 was depleted in these complexes, which was most readily evident in immunoprecipitates of PC12 cells. These reciprocal shifts in P23 and HOP are to be expected (38, 39). The HSP relay functions in a bidirectional manner; if folding is not accomplished, J domain proteins complexed to an HSP70 member and additional cofactors, such as CHIP, can redirect the client protein to degradation (30, 31). Inhibition of HSP90, however, increased the steady state level of the A_2A_ receptor. This suggests that the A_2A_ receptor can reach its active conformation without engaging HSP90α. We also used kifunensine and bortezomib to preclude access of the A_2A_ receptor to ER-associated degradation. These manipulations increased steady state levels of active A_2A_ receptors in PC12 cells by about 1.5-fold, but they only had a modest effect on the composition of A_2A_ receptor-HSP complexes that were recovered from PC12 cells. This indicates that under steady state conditions, the HSP machinery can cope with the increased anterograde flux of receptor. In contrast, treatment of HEK293 cells with kifunensine led to the accumulation of A_2A_ receptor complexes containing HSP90 and HSP70. This discrepancy can presumably be attributed to the higher expression level of the heterologously expressed receptor. Irrespective of these cell-specific differences, our observations highlight the importance of chaperoning a GPCR on the cytosolic side. HSP70 relies on HSP40, which is involved in recruiting unfolded and partially folded substrates to HSP70 (31). Our experiments failed to uncover the nature of the HSP40 member required for folding of the A_2A_ receptor. To the best of our knowledge, there is only one precedent of an HSP40 member (*i.e.* DRiP78 (dopamine receptor-interacting protein of 78 kDa)) that has been shown to regulate folding of GPCRs; the A_1_ and D_1_ dopamine receptors recruit DRiP78 during their maturation (40, 41). DRiP78 binds to the conserved hydrophobic motif (F/L)*X*_3/4_F*XXX*F, which is located in the C-terminal helix 8 of almost all rhodopsin-like GPCRs. Mutations in this motif inhibit ER export of the adenosine A_1_-, vasopressin V_1b_-, adrenergic α_2B_-, and angiotensin AT_1_ receptors (42–44). The (F/L)*X*_3/4_F*XXX*F is also present in the A_2A_ receptor. Truncation of the C-terminal tail of both the A_2A_ and the A_1_ receptor results in a decrease or even in a loss of their cell surface expression (14, 44). However, we failed to detect any interaction between DRiP78 and the A_2A_ receptor (not shown), possibly because the A_2A_ receptor lacks the palmitoylated cysteine at the end of helix 8 (5).

Membrane proteins are prone to aggregation because of their large hydrophobic surfaces. Accordingly, the ER has evolved an intricate machinery that safeguards against the escape of unfolded, partially folded, or conformationally unstable versions of membrane proteins. From a teleological perspective, it is therefore plausible that ER quality control must be stringent and prefer to err in favor of retention rather than allow a potentially dysfunctional membrane protein. This property combined with appropriate mutations can give rise to folding diseases, where a potentially functional protein is retained. The most prominent example is CFTRΔF508; a cytoplasmic chaperone relay system assists in quality control of CFTRΔF508 (45), and pharmacological manipulations of the relay system can in principle be employed to allow for escape of the protein to the cell surface (46). ABC transporters have large intracellular domains. Hence, cytoplasmic chaperones are readily envisaged to play a major role in sampling the conformational state of the nascent protein. However, more recently, cytoplasmic chaperones were shown to sense the folding state of the NaCl transporter, a membrane protein of the solute carrier-12 family (SLC12A3) (47). Transporters of the SLC family do not have large intracellular domains. Our findings that a cytoplasmic chaperone relay is recruited to the A_2A_ receptor are in line with the concept that the folded state must be sampled at the cytoplasmic side prior to recruitment of the SEC24/SEC23 dimer. Although this chaperone/COPII exchange model is presumably relevant to many different classes of membrane proteins, it is of particular importance to GPCRs. It may have repercussions because defective folding of GPCRs and ER retention is known to give rise to many different types of human diseases, ranging from retinal degeneration to metabolic diseases (for a review, see Ref. 48). In some instances, folding can be restored by adding a membrane-permeable ligand as a pharmacological chaperone (pharmacochaperone). GPCRs can be expressed in functional form in *E. coli* (*i.e.* they interact with their cognate G proteins) (49–51); however, active receptors do not accumulate to levels that can be achieved in mammalian cells, and a large fraction of the receptor protein forms aggregates. Pharmacochaperones fail to elicit their eponymous action (*i.e.* enhance the formation of active receptors) in *E. coli* (41). Both the low level expression and the ineffectiveness of pharmacochaperones are possibly related to the absence of the appropriate chaperone relay in bacteria. If pharmacochaperoning does rely on cytosolic chaperones, it may be enhanced by appropriate manipulations of the cytosolic chaperones to overcome obstacles inherent in pharmacochaperoning. This is best illustrated by considering mutations in the V_2_-vasopressin receptor that give rise to X-linked nephrogenic diabetes insipidus. Some of these can be rescued by pharmacochaperoning, such that the folded receptor appears at the cell surface (20). However, because the pharmacochaperones are antagonists, they block the receptor and thus render it unresponsive to the action of the endogenous agonist vasopressin. A compound that targets the chaperone relay may sensitize the ER-retained receptor to the action of low concentrations of pharmacochaperone that do not cause full blockage of the surface-resident receptor. This is currently being explored.

## Footnotes

3 The abbreviations used are:

GPCR: G protein-coupled receptor
CHIP: C terminus of HSP70-interacting protein
CGS21680: 3-(4-(2-(6-amino-9-((2*R*,3*R*,4*S*,5*S*)-5-(ethycarbamoyl)3,4-dihydroxyoxolan-2-yl)amino)ethyl)phenyl)propanoic acid
COPII: coat protein complex II
DDM: *n*-dodecyl-β-d-maltoside
17-DMAG: 17-dimethylaminoethylamino-17-demethoxygeldanamycin
ER: endoplasmic reticulum
HSP: heat-shock protein
MBP: maltose-binding protein
MBP-A_2A_-C-tail: a fusion protein comprising MBP and the C terminus of the A_2A_ receptor
TAP: tandem affinity purification
XAC: xanthine amine congener
ZM241385: 4-(2-(7-amino-2-(2-furyl)-(1,2,4)-triazolo-(2,3-*a*)-(1,3,5)-triazin-5-ylamino)ethyl)phenol)
TEV: tobacco etch virus.
